# Effectiveness of hypnosis for pain management and promotion of health-related quality-of-life among people with haemophilia: a randomised controlled pilot trial

**DOI:** 10.1038/s41598-019-49827-1

**Published:** 2019-09-16

**Authors:** Ana Cristina Paredes, Patrício Costa, Susana Fernandes, Manuela Lopes, Manuela Carvalho, Armando Almeida, Patrícia Ribeiro Pinto

**Affiliations:** 1 Life and Health Sciences Research Institute (ICVS), School of Medicine, University of Minho, Braga, Portugal; 2 ICVS/3B’s – PT Government Associate Laboratory, Braga/Guimarães, Portugal; 3 Faculty of Psychology and Education Sciences, University of Porto, Porto, Portugal; 4 Centre of Hemophilia, Department of Transfusion Medicine and Blood Bank, São João University Hospital Centre, Porto, Portugal

**Keywords:** Quality of life, Clinical trials, Outcomes research

## Abstract

Joint deterioration and associated chronic pain are common among people with haemophilia (PWH), having an impact on quality-of-life. Though non-pharmacological strategies are recommended, psychological interventions to promote pain control and quality-of-life have scarcely been tested in haemophilia. This randomised controlled pilot trial aimed to assess feasibility, acceptability and effectiveness of hypnosis for pain management and promotion of health-related quality-of-life (HRQoL) among PWH. Twenty adults were randomised either to four weekly hypnosis sessions plus treatment-as-usual (experimental group; EG) or treatment-as-usual only (control group; CG). Participants completed sociodemographic and clinical assessment, measures of pain, HRQoL and emotional distress before (T1) and after (T2) intervention. Changes were analysed by calculating the differences between T1 and T2, and the groups were compared through independent-sample t tests (or chi-squared). Retention rates (90%) and analysis of patient satisfaction showed good acceptability and feasibility of the intervention. The EG (n = 8) had a higher reduction on pain interference than the CG (n = 10) (*d* = −0.267). A higher improvement on HRQoL (EQ-5D index: *d* = 0.334; EQ-5D VAS: *d* = 1.437) and a tendency towards better haemophilia-related quality-of-life (A36-Hemofilia QoL) were also evident in the EG. This is the first study showing the effectiveness of hypnosis to reduce pain interference and promote HRQoL among PWH.

## Introduction

Haemophilia is an X-linked rare bleeding disorder, resulting from a deficiency in blood coagulation factor VIII (haemophilia A) or IX (haemophilia B), and characterized by a pattern of spontaneous bleeding episodes that most often occur in the muscles (haematomas) or joints (haemarthrosis)^1^. The frequency of spontaneous bleeds is associated with disease severity, which is classified according to plasma levels of clotting factor activity (severe: <1% of normal factor level; moderate: 1 to 5%; mild: 5 to 40%)^2^. Intravenous administration of factor replacement concentrate is the mainstay of treatment, delivered to stop bleeds (on-demand) or to prevent its occurrence (prophylaxis)^3^. Prophylaxis can potentially prevent bleeds altogether, but its use as a routine treatment since childhood has only been considered a standard of care during the last two decades. As a consequence, most adults with severe haemophilia did not benefit from prophylaxis while growing up and are now afflicted with some degree of joint damage, due to frequent haemarthrosis and lifelong accumulation of intraarticular blood^4^.

Haemarthrosis are painful events that, if repeated, progressively contribute to the development of chronic haemophilic arthropathy, characterized by irreversible joint damage, disability and chronic pain^5^. Pain is therefore a very relevant issue among people with haemophilia (PWH), with studies reporting a prevalence of chronic pain ranging from 35 to 66%^6–9^, and about half the participants in some investigations complaining of daily arthritic pain^10,11^. In addition, it has been demonstrated that PWH who experience more intense or frequent pain have lower quality-of-life^8,10,12,13^, further highlighting the unquestionable relevance of an adequate pain management in this field. Yet, haemophilia-related pain is still under-recognized and suboptimally treated^14,15^, and several authors have called on the need to improve its assessment and management, advocating for a combination of pharmacological and non-pharmacological techniques^7,16,17^.

Besides clinical variables (*e*.*g*. bleeding rate, joint status, pain), health-related quality-of-life (HRQoL) is a valuable outcome of research to capture patients perspective on their health. This is even more relevant in such burdensome conditions like haemophilia, with lifelong complications and demanding treatments^18^. Indeed, research on this topic has shown that PWH have lower HRQoL than the general population^12,19–21^. This has contributed to current haemophilia guidelines, which emphasize quality-of-life promotion as a relevant endpoint of comprehensive care^22^. However, the few interventional studies assessing HRQoL outcomes among adult PWH have mainly focused on the effects of physical exercise and physiotherapy interventions^23,24^, or on the impact of different factor replacement treatment modalities^25,26^. To our knowledge, the efficacy of psychological interventions specifically aiming at improving pain management and quality-of-life among PWH has never been tested, leaving a significant gap in evidence-based treatments targeting these outcomes.

The existing research focusing on psychological interventions among PWH is outdated and has focused mainly on hypnosis, showing positive results for disease management^27–29^. Indeed, the medical use of hypnosis is approved since 1955 by the British Medical Association^30^ and is recognized as an effective intervention to help manage chronic pain^31^, also contributing to improve HRQoL across distinct medical fields^32–34^. In haemophilia, the promising results that have been reported using hypnosis make the discontinuity of this line of research somewhat surprising, and thus encourage the development of investigations to further explore its usefulness.

The aim of this work was to assess the effectiveness of a hypnosis intervention for pain management and promotion of HRQoL among people with haemophilia. Since this is a rare disease, with an estimated number of 700 cases in Portugal^35^, we conducted a pilot trial to address if a future randomised controlled trial (RCT), with a larger sample, would be feasible and well-accepted by patients.

## Results

### Feasibility and acceptability

**Figure 1:**
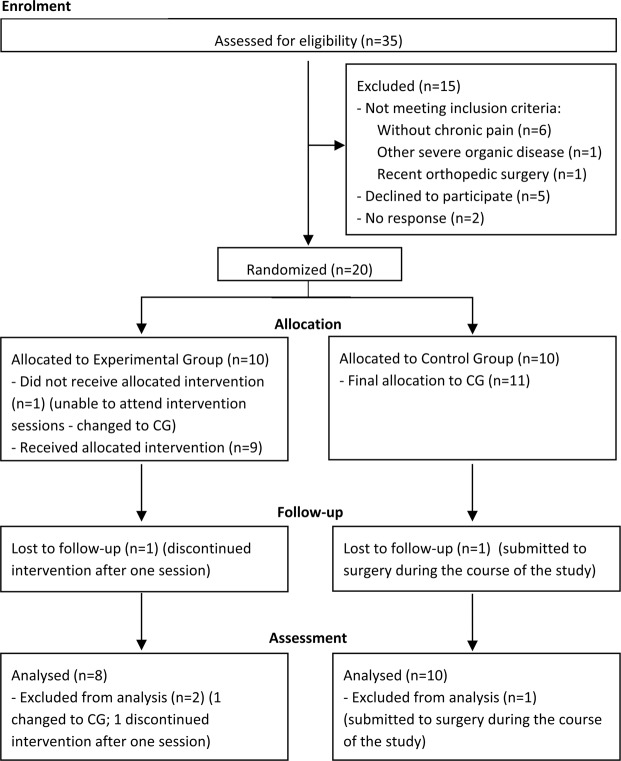
Flowchart of study participants.

Patient screening and assessment (pre and post-intervention) occurred from January to June 2018. The flow of patients through the study, reasons for ineligibility and refusals are shown in Fig. 1. Thirty-five adult patients with haemophilia were identified as eligible. Of these, 20 accepted to participate and met the inclusion criteria, and were therefore randomised to one of the study groups. One participant from the CG was excluded because he underwent orthopaedic surgery during the duration of the trial, and one participant in the EG dropped out of the intervention after one session due to unavailability to attend the remaining sessions. Another participant who had been initially allocated to the EG also expressed unavailability to attend the intervention sessions, but was willing to change to the CG. Thus, the final analysed sample included 8 participants in the EG and 10 participants in the CG.

Retention rate in the pilot trial was 90%, with participants in the EG (n = 8) attending all hypnosis sessions. Concerning satisfaction with intervention, 2 (25%) participants in the EG revealed to be “satisfied” and 6 (75%) “very satisfied” with the intervention. All participants (8, 100%) stated they felt “a lot” better after the four sessions, said they “agree” that these interventions would be useful in haemophilia centres and also “agree” that they would wish to participate in further sessions, if available.

No unanticipated harm or detrimental effects of intervention were reported.

### Sociodemographic and clinical characteristics

Baseline sociodemographic and clinical participant characteristics are summarized in Table 1. The groups were not statistically different on any of these variables, although a large effect size (ES) was found for the differences on age (*d* = −0.879) and number of affected joints (*d* = −0.985); and a medium ES for the number of painful locations (*d* = −0.770).

**Table 1:** Characteristics of study participants and differences between groups at baseline.

	Total Sample (N = 18)	Control Group (n = 10)	Experimental Group (n = 8)	t/χ^2^ (df)	p	*d*/φ
**Sociodemographic**						
Age	45.00 (9.48)	41.70 (10.41)	49.13 (6.62)	−1.748 (16)	0.100	−0.879
Education (≥12 years)	9 (50%)	5 (50%)	4 (50%)	0.000 (1)	1.00	0.000
Marital status (married)	12 (66.7%)	7 (70%)	5 (62.5%)	0.113 (1)	0.737	0.079
Prof. status (employed)	11 (61.1%)	6 (60%)	5 (65.5%)	0.012 (1)	0.914	0.026
**Clinical**						
Haemophilia A	15 (83.3%)	9 (90%)	5 (75%)	0.720 (1)	0.396	0.200
Severe haemophilia^a^	14 (77.8%)	7 (70%)	7 (87.5%)	0.787 (1)	0.375	0.209
Prophylaxis	7 (38.9%)	4 (40%)	3 (37.5%)	0.012 (1)	0.914	0.026
Inhibitors	2 (11.1%)	0	2 (25%)	2.813 (1)	0.094	0.395
Bleeds last month (yes)	8 (44.4%)	3 (30%)	5 (62.5%)	1.901 (16)	0.168	0.325
Number of affected joints	5.44 (2.99)	4.30 (2.83)	6.88 (2.696)	−1.958 (16)	0.068	−0.985
Target joint^b^	13 (72.2%)	7 (70%)	6 (75%)	0.055 (1)	0.814	0.055
**Pain**						
Duration ≥20 years	13 (72.2%)	7 (70%)	6 (75%)	0.055 (1)	0.814	0.055
Painful locations last year	4.11 (1.97)	3.50 (1.58)	4.88 (2.23)	−1.531 (16)	0.145	−0.770

Note: Continuous variables are presented as mean (standard deviation) and categorical variables are presented as n (%).

^a^Severe: factor level < 1% of normal, Moderate: factor level 1 to 5% of normal^2^.

^b^Three or more spontaneous bleeds into a single joint within a consecutive 6-month period^2^.

No statistically significant differences were found between the groups at baseline on the analysed outcomes, except on the “treatment difficulties” subscale of the A36 Hemofilia-QoL (t_(16)_ = 2.52; p = 0.023; *d* = 1.27) (results not shown). This subscale score revealed higher difficulties for the EG.

### Comparison of pre/post-intervention changes among groups

Table 2 shows the differences from baseline to post-intervention in each outcome for each group, comparing them. Concerning work absences, the number of days missed from work in the previous month decreased in the EG (Δ = 5.80 days; SD = 13.57) and had a slight increase in the CG (Δ = −0.83; SD = 2.04), corresponding to a medium ES (*d* = −0.656).

**Table 2:** Baseline and post-intervention scores, and results of the independent samples t test for pre/post-intervention differences (Δ) between study groups.

	Control Group (n = 10)		Experimental Group (n = 8)		Δ (T1-T2)		Test statistics		
	Baseline M (SD)	Post-intervention M (SD)	Baseline M (SD)	Post-intervention M (SD)	ΔCG M (SD)	ΔEG M (SD)	t (df)	p	*d*
No. missed work days (last month)^a^	0	0.83 (2.04)	7.00 (13.04)	1.2 (1.79)	−0.833 (2.04)	5.80 (13.57)	−1.083 (4)	0.338	**−0**.**656**
No. bleeds last month	0.90 (1.91)	1.20 (1.40)	1.25 (1.17)	1.50 (1.69)	−0.30 (1.42)	−0.25 (1.83)	−0.065 (16)	0.949	−0.031
Pain intensity	4.27 (1.77)	3.81 (1.51)	4.22 (1.99)	4.13 (1.09)	0.46 (2.11)	0.09 (1.48)	0.412 (16)	0.686	0.195
Pain interference	3.05 (1.58)	2.64 (1.72)	3.96 (2.62)	3.21 (2.30)	0.41 (0.99)	0.75 (1.53)	−0.563 (16)	0.581	**−0**.**267**
EQ-5D-5L: VAS	62.5 (13.99)	55.00 (13.33)	56.25 (21.17)	70.00 (18.89)	7.50 (17.20)	−13.75 (10.94)	3.029 (16)	0.008	**1**.**437**
EQ-5D-5L: Index score	0.70 (0.08)	0.74 (0.08)	0.58 (0.23)	0.68 (0.09)	−0.04 (0.12)	−0.09 (1.48)	0.703 (16)	0.492	**0**.**334**
A36 Hemofilia-QoL: global score	99.7 (13.06)	108.9 (13.83)	92.38 (28.25)	107.13 (23.27)	−9.20 (8.87)	−14.75 (20.78)	0.706 (9)	0.498	**0**.**335**
Physical health [0–32]	19.90 (2.52)	21.80 (4.29)	18.75 (6.88)	22.38 (4.63)	−1.90 (5.20)	−3.63 (6.55)	0.624 (16)	0.541	**0**.**296**
Daily activities [0–16]	10.00 (3.59)	12.40 (3.63)	7.38 (6.23)	12.38 (4.07)	−2.40 (3.47)	−5.00 (5.50)	1.225 (16)	0.238	**0**.**581**
Joints [0–12]	7.50 (2.17)	6.90 (1.85)	6.88 (1.73)	7.00 (3.16)	0.60 (2.32)	−0.13 (2.59)	0.626 (16)	0.540	**0**.**297**
Pain [0–8]	4.30 (2.11)	5.20 (1.40)	4.50 (1.07)	5.13 (1.36)	−0.90 (2.28)	−0.63 (2.07)	−0.265 (16)	0.795	−0.126
Treatment satisfaction [0–8]	6.40 (1.58)	6.60 (1.08)	6.88 (1.13)	6.75 (0.89)	−0.20 (1.23)	0.13 (0.99)	−0.606 (16)	0.553	**−0**.**288**
Treatment difficulties [0–16]	14.60 (1.58)	13.70 (3.16)	11.28 (3.85)	12.75 (2.38)	0.90 (2.88)	−1.50 (2.73)	1.797 (16)	0.091	**0**.**852**
Emotional functioning [0–20]	12.20 (3.55)	15.90 (2.89)	13.13 (4.91)	15.50 (3.21)	−3.70 (2.95)	−2.38 (4.34)	−0.771 (16)	0.452	**−0**.**366**
Mental health [0–12]	8.50 (2.55)	9.20 (2.20)	8.00 (3.21)	8.50 (3.78)	−0.70 (3.83)	−0.50 (1.60)	−0.138 (16)	0.892	−0.066
Relationships and social activity [0–20]	16.30 (2.91)	17.20 (1.62)	15.63 (5.29)	16.75 (6.32)	−0.90 (2.77)	−1.13 (3.27)	0.158 (16)	0.876	0.075
HADS: Depression	0.60 (1.07)	0.50 (0.71)	1.50 (3.85)	1.50 (3.85)	0.10 (1.29)	0 (0.53)	0.205 (16)	0.840	0.097
HADS: Anxiety	3.50 (2.88)	3.60 (2.55)	3.38 (2.56)	3.50 (3.16)	−0.10 (2.99)	−0.13 (2.42)	0.019 (16)	0.985	0.009

^a^Only assessed among employed participants (CG: n = 6; EG: n = 5).

Note: Possible score ranges for each of the A36 Hemofilia-QoL subscales are specified in square brackets.

Abbreviations: VAS, Visual Analogue Scale; HADS, Hospital Anxiety and Depression Scale.

Bold font indicates an effect size >0.20.

Regarding the clinical parameters under assessment, pain interference yielded significant results (*d* = −0.267), decreasing in both groups, but with a higher reduction in the EG (Δ = 0.75; SD = 1.53) than in the CG (Δ = 0.41; SD = 0.99). The differences on pain intensity from pre to post intervention were not significant (*d* = 0.195) between the groups.

In what concerns general HRQoL, there was a statistically significant difference between groups on EQ-5D VAS (t_(16)_ = 3.03, p = 0.008, *d* = 1.437), with a mean 13.75 (SD = 10.94) point improvement in the EG and a mean 7.50 (SD = 17.20) decrease in the CG. EQ-5D index scores increased in both groups, yet with a slightly higher improvement in the EG (small ES, *d* = 0.334). Concerning haemophilia-related quality-of-life, the EG revealed a global tendency towards better HRQoL at post-intervention, with a higher difference (larger improvements) than the CG on the global score (ES = 0.335) and in 4 of the 9 specific dimensions. The higher effect sizes for these improvements in the EG were found on “treatment difficulties” (large ES, *d* = 0.852) and “daily activities” (medium ES, *d* = 0.581). Despite having small effect sizes, “physical health” (*d* = 0.296) and “joints” (*d* = 0.297) also yielded higher improvements in the EG, compared with the CG. Finally, the magnitude of the differences concerning depression and anxiety was not significant (depression, *d* = 0.097; anxiety, *d* = 0.009).

## Discussion

This manuscript reports a randomised controlled pilot trial aiming to assess the feasibility, acceptability and effectiveness of a hypnosis intervention among people with haemophilia (PWH). Findings revealed, for the first time, the specific benefits of hypnosis in diminishing pain interference and in improving health-related quality-of-life (HRQoL) among PWH.

Important practical insights were also retrieved from this study, namely concerning the feasibility of a future randomized controlled trial (RCT). Considering the rarity of this disease and the inclusion criteria for participation, the main challenge in implementing a future RCT would be the recruitment of a big enough sample size to guarantee statistical power. In this case, implementing a multi-centre trial could be a potential solution. From this pilot study, it also became clear that time availability to participate in the intervention sessions is a relevant constraint, particularly for participants who have a full-time occupation. In this sense, it seems that accounting for individual allocation preferences could be advantageous to guarantee bigger sample sizes, namely by adopting patient preference designs. In these, participants with a strong preference for one of the arms are assigned to it, while the others are randomised between the groups. This approach contributes to improve recruitment rates and is more advantageous when active involvement of patients is warranted, without compromising internal and external validity^36,37^.

Globally, this study showed that it is feasible to develop and implement an RCT with PWH that is based on hypnosis. The participants in the experimental group (EG) attended all sessions (except one person that discontinued the intervention after the first session and was thus excluded from the study), demonstrating good acceptability of the intervention. All participants reported to be satisfied with the intervention and expressed the wish to keep attending, if more sessions were available.

Former studies have previously reported the implementation of hypnosis among PWH, with positive results in controlling bleeding, reducing pain and alleviating stress^27–29^. Other investigations, describing imagery and relaxation techniques similar to hypnosis, have also achieved reductions in arthritic pain for PWH undergoing intervention^38,39^. These were encouraging results for the use of hypnosis as an adjunctive strategy for haemophilia care, but the fact that most conclusions were based on case studies and lacked a rigorous methodology warrants further research to establish more definitive conclusions^40^. Surprisingly, the last reports of studies analysing the efficacy of hypnosis in the haemophilia field date from the early 90’s. To our knowledge, no more recent research was conducted on this subject, hence the relevance of further exploring this issue.

Previous research has described reductions on pain intensity following hypnosis across a variety of other chronic pain conditions^41–48^. Nevertheless, and despite the good results yielded by the present study on pain interference, similar improvements on pain intensity were not found. One possible explanation for this result might be the long duration of pain within this sample of patients. As far as we are aware, there are no studies investigating hypnosis effectiveness among patients with such a long pain duration as in this pilot trial, with around 70% of the participants having pain for over 20 years. In the literature reviewed on this subject, the highest mean pain duration identified was 13 years^47^. This is particularly significant since continuous and prolonged painful inputs from the damaged joints may cause an amplification of the responses of peripheral nociceptors (peripheral sensitization) and central nervous system neurons (central sensitization), resulting in an over activation of the nociceptive system^49^. Indeed, central and peripheral sensitization have been demonstrated in PWH^17^, and since the level of sensitization is dependent on pain duration^50^, these phenomenon may help to understand the non-significant changes in pain intensity found in the present study. In addition to physiological mechanisms, cognitive and emotional factors could also help explain the results, as these also play an important role in pain modulation^51^. Future trials with adequate sample sizes should therefore control for the potential moderator effect of psychological variables on intervention outcomes.

Additionally to a very long pain duration, it is possible that 4 sessions of hypnosis might have been insufficient to produce a significant decrease on pain intensity. Although some studies have achieved positive results with only 3 or 4 sessions^41,42,45^, most chronic pain trials include more sessions. It is true that brief interventions have been proven effective in conditions such as surgical acute pain^52^, but it is likely that the specificities of chronic pain demand a higher number of sessions. For example, Tan *et al*.^43^ showed that 2 self-hypnosis training sessions were effective in reducing pain intensity and interference, but these were followed by six weekly reminder telephone calls that possibly accounted for the positive results. Indeed, it has been recommended that a full hypnosis treatment should comprise 8 or more sessions^53^, and the question of the ideal number of sessions to achieve a clinically significant improvement in symptoms should be further analysed in future studies. Lastly, and given the low sample size, it is also important to consider the contribution of individual results for the group averages^54^. In this scope, it should be noted that one participant in the hypnosis sessions had a fall during the treatment period, with a later X-ray revealing a displacement of his knee prosthesis, which understandably influenced pain intensity ratings increase between pre and post-test.

Though pain intensity is traditionally the most valued outcome of pain research, it is also important to analyse the effects of treatments on other pain-related outcomes, to shed a more comprehensive light over the burden of pain^48^. Corroborating other reports^42,43^, the current study suggests that hypnosis intervention is effective in decreasing the perception of pain interference. Though there was a reduction on pain interference in both groups, it was higher in the EG, accounting for a significant effect size. The relevance of this finding is underscored by the high prevalence of pain in haemophilia and the recognition that it can be highly disruptive, particularly for patients with chronic pain^8^. Indeed, pain has been associated with lower HRQoL among PWH^8,10,12,13^, yielding a global negative impact on daily life. For instance, an international survey reported that as much as 89% of the participants had pain that interfered with daily activities^12^, mainly on physical functioning domains, but also having an impact on mood and enjoyment of life^55^. Congruently, previous findings of our team have shown that the greatest pain interference reported by Portuguese PWH was on dimensions related to daily activities, such as work and mobility, but also on mood^13^. In this scope, a positive association between pain interference and distress was also found among PWH, heightening the relevance of interventions that target pain interference in this population^56^. This body of evidence and our current findings add to recent publications calling for an improvement on pain management among PWH, namely through a broader adoption of non-pharmacological strategies^7,14,16,17^. Given the acknowledged negative impact of pain and these recent calls to improve its management in the haemophilia field, we would advocate for an urgent shift on research, from collection of observational data towards the implementation and testing of pain management interventions.

Notwithstanding the encouraging results showing reductions on pain interference, the most notable findings from this intervention concern HRQoL. In this sample, baseline EQ-5D VAS scores were lower (worse perception of health status) than those described in other haemophilia studies^8,20,25^. However, the effect of age has to be considered when comparing these figures, since those studies included data from younger samples (mean ages ranging from 29.7 to 37 years old). Research has indeed demonstrated that HRQoL scores tend to be lower among older PWH^12,19,57^, which has been at least partially explained by increased joint deterioration in this age group^19,57^. Congruent data was found in the present sample, with participants in the EG having a higher mean age and more affected joints. Nonetheless, these participants had a higher increase in EQ-5D index scores than those in the CG and an unequivocal improved assessment of their overall health status (EQ-5D VAS) at post-test, with participants in the CG even presenting a reduction in EQ-5D VAS scores. This is even more noteworthy considering the older mean age of participants in the EG, suggesting that hypnosis is an effective way to improve the subjective evaluation of health, even among older PWH who present more joint deterioration.

Concerning haemophilia-related quality-of-life, the improvements were higher for the EG on most dimensions, with the largest effect sizes on global HRQoL, “daily activities”, “treatment difficulties”, “physical health” and “joints”. These data show a more marked benefit of the intervention on dimensions related to the perception of physical health, which indeed tends to be more affected in PWH, comparing to mental health dimensions^21,55^. In this scope, it is also relevant to note that there were no significant reductions on “pain” scores (A36 Hemofilia-Qol). However, the 2 items composing this subscale refer only to pain frequency, which was not a specific target of the intervention sessions. Therefore, no changes were expected in this dimension.

As outlined in the introduction section, there are no published controlled trials specifically aiming to test psychological interventions that promote HRQoL among adult PWH, despite the acknowledgment that it is impaired in these patients^12,19–21^. Only one of the former reports of hypnosis in haemophilia makes a reference to quality-of-life, affirming that it is improved among patients using self-hypnosis, though it is not clear whether it was formally assessed^29^. Despite the absence of interventional studies with PWH, some comparisons can be drawn from investigations analysing the utility of hypnosis in other conditions that, like haemophilia, are associated with disabling symptoms. Corroborating the findings from this paper, those have shown that hypnosis is effective in improving HRQoL among patients with irritable bowel syndrome^32^, fibromyalgia^33^ and breast cancer^34^. This body of evidence further supports the utility of hypnosis to promote HRQoL and favours the pursuit of this line of research, encouraging the adoption of strategies that can improve the well-being, and thus the quality-of-life, of patients with chronic conditions.

Lastly, it is also relevant to note that the present results revealed a reduction in work absences in the EG, which could be related to the significant findings concerning global pain interference. Also, fewer missed days from work could be associated with the improved perception of HRQoL among participants in the EG.

This investigation has some limitations to consider. Although this was a pilot study, the small sample size hinders the establishment of definitive conclusions concerning the effectiveness of the intervention, which should be overcome in a future, adequately powered RCT. Additionally, specific measures should be adopted to avoid treatment contamination between the groups (*i*.*e*. the possibility that participants in the EG may disseminate information concerning the intervention to participants in the CG). However, the significant effect sizes obtained indicate treatment effectiveness and cannot be dismissed as a promising research direction^58^. In addition, the demographic and clinical differences between groups at baseline may have influenced the outcomes. Namely, patients in the EG have more affected joints and more painful locations which, as discussed above, can impact HRQoL and pain experience. Similarly, it would also be relevant to consider the role of other clinical variables, such as prophylaxis status or inhibitor development, and to control for their influence on the studied outcomes.

Another important variable to account for in a future RCT would be hypnotic susceptibility, since it has been suggested that highly susceptible individuals derive increased benefits from hypnotic pain interventions^59^. In this scope, it might be plausible that hypnotic susceptibility levels could at some degree account for the lack of a significant decrease on pain intensity levels in this study. However, it has also been argued that, in clinical settings, the success or failure of hypnotic interventions is not dependent on hypnotisability, which would only explain a small percentage of variance in outcomes^60^. Studies with chronic pain patients have reached inconsistent conclusions on this matter, with some findings revealing increased reductions on pain intensity scores among highly susceptible individuals^46^, others concluding that susceptibility was not related to outcomes^43–45,48^, and one study even reporting an inverse relationship between hypnotic susceptibility and reduction on pain intensity^42^. Undoubtedly, the inconsistent findings on this matter^54,60^ and the potential impact of hypnotic susceptibility on intervention outcomes justify the need to consider its inclusion as a potential moderator, or as a criteria for group stratification, in hypnotic intervention studies^58^.

Another suggestion for further studies would be a thorough record of self-hypnosis teaching and home practice, as a possible mean to explain clinically significant changes and long-term results. Also relevant to consider is the inclusion of an active control group besides the treatment-as-usual condition (*e*.*g*. attention group, relaxation training), in order to compare the relative efficacy of hypnosis and to rule out placebo effects as an explanation for the results obtained.

In conclusion, this pilot trial showed that it would be feasible to conduct an RCT to analyse the effectiveness of a hypnosis intervention among PWH. This was, to our knowledge, the first study suggesting that hypnosis is effective in decreasing pain interference and in improving the perception of HRQoL among PWH, providing a promising research direction that should be replicated with larger sample sizes and more homogenous groups, in order to establish more definitive conclusions.

## Methods

### Design

This was a two-arm randomised controlled pilot trial, with one experimental (intervention) group (EG) and one control group (CG), and three assessment moments: baseline (before intervention), post-test (after intervention) and 3 months after intervention. This manuscript reports the results from baseline (T1) and post-test (T2) assessment moments.

This investigation was approved by the Portuguese National Data Protection Agency, the Life Sciences and Health Ethics Subcommittee (University of Minho) and the Ethics Committee from São João University Hospital Centre —E.P.E.. All procedures were carried out in accordance with the Declaration of Helsinki and relevant guidelines for clinical research with human participants, and reported following the Consolidated Standards of Reporting Trials (CONSORT). This pilot trial is part of a larger study, registered at clinicaltrials.gov (NCT02870452) on August 17^th^, 2016.

### Participants

Participants were recruited from the European Haemophilia Comprehensive Care Centre (EUHANET certified centre) and Reference Centre for Congenital Coagulopathies (certified by the Portuguese Health Ministry) at São João University Hospital Centre, Porto, Portugal. Eligible patients were identified and approached by the clinicians of the haemophilia centre to be referred to the investigators, who made a later telephone call for a more comprehensive evaluation of inclusion criteria. These included (a) male gender; (b) age ≥18 years; (c) moderate or severe haemophilia A or B, with or without inhibitors; (d) chronic pain (as defined by the European Haemophilia Therapy Standardisation Board^7^; and (e) ability to consent voluntary participation to the study. The exclusion criteria were (a) severe and debilitating neurological conditions (*e*.*g*., dementia); (b) severe organic or psychiatric conditions (*e*.*g*., cancer, schizophrenia); (c) undergoing any form of psychotherapy at time of enrolment; (d) having had orthopaedic surgery less than six months before study start or having it scheduled for the following six months; and e) unavailability to commit to four weekly intervention sessions.

During a visit to the haemophilia centre, participants signed the informed consent, performed baseline assessment and were randomly allocated either to intervention or control group. At this time, the four weekly intervention sessions were scheduled for participants in EG. Five weeks after baseline all participants completed the post-test assessment.

Since this was a pilot study, a formal sample size calculation was not performed.

### Hypnotic intervention

Patients in the EG attended four consecutive weekly individual 60-min hypnotic sessions while maintaining treatment-as-usual. The hypnotic scripts were adapted from different sources^61–65^ and the intervention was conducted verbatim by one of the authors (PRP), who is a PhD Clinical Health Psychologist with certified advanced training in clinical hypnosis.

In order to engage patients in hypnosis, the first step is to explain its principles, providing patients with a rationale for its learning and use. In all sessions, theoretical-educative contents were provided and discussed before hypnotic induction. These focused on pain education, namely through the use of metaphors based on the gate control theory, emphasizing that pain results from a complex and dynamic interplay between biological processes and psychological factors (cognitive and emotional), as well as on contents regarding the relaxation response.

The hypnosis sessions comprised the following stages: (1) introduction/preparation of the patient (explaining the rationale underlying hypnosis, including dispelling potential myths, misconceptions and doubts; this step only in the first session); (2) hypnotic induction (suggestions to promote a state of relaxation and focused awareness); (3) deepening procedure (further suggestions for achieving a more deeply relaxed and focused state); (4) symptom-specific therapeutic suggestions (specific for haemophilia, aiming to change or improve symptoms and/or maladaptive behaviors, such as pain); (5) posthypnotic suggestions (to extend the benefits obtained beyond the session setting) and, finally (6) “waking up” the patient. On the first session, all the patients were taught self-hypnosis^61^ in order to be provided with means to perform hypnosis independently by themselves, thus reinforcing the posthypnotic suggestions. Self-hypnosis constitutes a powerful resource that guarantees the practice of the technique, independently and in an autonomous fashion, thereby empowering patients and giving them a sense of control and mastery over their problems and their lives^53,63^.

Regarding the hypnotic scripts, these consisted of an initial standardized hypnotic induction procedure (“Hartland’s Progressive Relaxation”)^64^, embracing suggestions to eye closure, concentration on breathing and promotion of a state of relaxation and focused awareness. It was then followed by the deepening procedure entitled “Special-Place Deepening Technique”^65^. This technique was directed to the selection of a comfortable and peaceful place, according to the personal preference of each participant (*e*.*g*. beach, garden, an autobiographical event…), wherein patients are suggested to experience several sensorial details of the place, along with allusions to safety and control. After induction and deepening procedures, specific hypnotic techniques were administered. In the first session, the “Healing White Light”^64^ technique was used to promote natural healing of the body against haemophilia usual symptoms (swellings, bleeds, bodily pains), intending to induce a general sense of well-being and health fitness. In the remaining three sessions, the “Pain Switch” technique, along with “Direct Therapeutic Suggestions For Pain Control” were employed^61^. All sessions included the “Ego Strengthening Technique”, which is potentially powerful because it increases the patients’ ability to access inner resources, improving their stress management skills and fostering a sense of control over the illness^65^. All suggestions were made on a repetitive basis at each session and all sessions ended with post-hypnotic suggestions before waking up the patient. These underscored that any experience of well-being, healing and comfort obtained would remain with the patient and last beyond the sessions, becoming a permanent part of how the patient lives and copes with disease and problematic issues.

An overview of the sessions, with further description of the techniques, is in Table 3.

**Table 3:** Overview of the hypnosis sessions and brief description of the techniques. Adapted from Hammond^61^, Jensen^62,63^, Heap & Aravind^64^ and Frederick & McNeal^65^.

**SESSION I**
**Introduction/Preparation of the patient**
Explanation of the rationale underlying hypnosis, including dispelling potential myths, misconceptions and doubts.
Theoretical-Educative Contents
**Pain Education: using the Gate Control Theory of pain**, **by Melzack and Wall (1965)** Transmission of information to educate patients about the subjectivity and multidimensionality of pain experience, conceptualizing it as the result of a dynamic and complex integration and interaction of psychological, biological and social dimensions. Emphasis on psychological factors as playing a critical role in pain experience, thus providing patients with a rationale for the importance of undergoing hypnosis, in order to become active resourceful agents, capable of self-managing their symptoms.
**Rationale underlying the Relaxation and the Stress Response** Explaining the relaxation response as a psychophysiological state opposite to the fight-or-flight response, characterized by the reduction of sympathetic/HPA activities and increases in the parasympathetic tone, neutralizing the excessive stress and inflammation. Focus on the physiological differences between the relaxation and the stress response.
**2**. **Hypnotic Techniques**
**2**.**1**. **Hypnotic Induction**
**Hartland’s Progressive Relaxation** Suggestions to eyes closure, concentration on breathing, promoting a state of relaxation and focused awareness, imagining oneself as being in an agreeable and comfortable place. Patients are asked to relax their muscles one by one (feet, calves, thighs, stomach, chest, back, neck, shoulders, arms, forearms, hands, eyebrows, eyes, jaw) and to be aware of proprioceptive and interoceptive sensations.
**2**.**2**. **Deepening Procedure**
**Special-Place Deepening Technique** Use of a metaphor of “descending a staircase” to help patients feel more deeply relaxed and hypnotized, by counting down from 10 to 1, which leads to a comfortable place according to the individual preference of each participant (*e*.*g*. beach, garden, playing, an autobiographical event…). Perceptions of colours, shapes, sounds, smells and kinaesthetic sensations are integrated into the suggestions, in order to make use of as many sensorial modalities as possible. Suggestions of feelings of calm, safety, peace of mind and inner strengths are given and associated with the image of being in the comfortable place.
**2**.**3**. **Therapeutic Suggestions**
**The Healing White Light** The patient is given suggestions to imagine a white light that is fiery and growing, moving around the body in order to induce a general sense of well-being and foster the process of healing. A special emphasis is placed on the joints more prone to hemarthrosis. It is intended to give the patient a sense of control over the illness as the white light travels through the body, infusing every part, eliminating the toxins and wastes, while returning a sense of purification and good health to the body. The script ends with a very positive note, emphasizing words such as vitality and energy, encouraging and giving hope to the patient (*e*.*g*. *“…and now…I want you to bring the sphere of white light…the powerful white light to your joints …allow the white light to settle in your joint ankles… knees…in your joint hips… elbows… and… it sends rays of healing white light…all over your joints…enclosing…enveloping your joints in a blanket of healing white light…a nice healing therapeutic warmth…and…the rays of healing white light spreads…all over your body…you feel the warm energy…flowing in your body…rays of energy flowing out from the sphere of white light…*).
**Ego Strengthening Technique** Aims to infuse the patient with a sense of competency, to enhance individual coping skills and to empower the belief in personal capabilities, activating deep internal healing powers. Develops the ability to access inner resources and activate internal survival mechanisms, aiming to increase confidence, self-esteem and self-efficacy. Promotes stress management skills as well as coping with haemophilia symptoms and constraints. This strategy builds up strength and resiliency, by reminding the patients of their strength, their ability to take control, and that taking control is their major strength (*e*.*g*. *“… feeling so good about how in control you are… of your body… your health… your life*.*”*).
**2**.**4**. **Post-Hypnotic Suggestions**
Aims to anchor the suggestions provided during the sessions, highlighting that any positive responses occurring during hypnosis should last beyond the session and become “permanent and automatic” (*e*.*g*. *“All benefits and skills that you have obtained from the session today…can become… more and more… a permanent part of how your brain works…what your brain is learning*, *and to the extent that it brings you comfort and a greater sense of control*, *then*, *is becoming more and more a permanent part of who you are… of how your brain works*.*”*).
**3**. **Teaching Self-Hypnosis**
The patient is instructed and taught to practice hypnosis on his own *(e*.*g*. *Whenever you wish to use hypnosis for yourself… all you need to do is find a comfortable place where you can sit or lie down*, *close your eyes and consciously relax your body… Each time you use your self-hypnosis*, *you will find yourself becoming more relaxed and calm in everyday situations*.*”)*.
**4**. **Waking up**
By counting upwards from 1 to 10, the patient is instructed to become increasing alert and to open the eyes (*e*.*g*. *“… when you open your eyes*, *you will be fully alert*, *fully oriented to your surroundings*, *and ready to carry on with the rest of the day*. *Any normal feelings*, *such as a bit of stiffness or tiredness*, *will quickly pass once you have taken a deep breath and had a good stretch*, *and you will find that you will feel the benefits of relaxing like this for the rest of the day*, *feeling alert*, *relaxed and refreshed*. *So*, *I’ll now count upwards: 1… 2… 3… 4… 5… 6… 7… 8… 9… 10… alert*, *wide awake and refreshed!”).*
**SESSION II**
**1**. **Theoretical-Educative Contents**
**2**. **Hypnotic Techniques**
**2**.**1**. **Hypnotic Induction: “**Hartland’s Progressive Relaxation”
**2**.**2**. **Deepening Procedure: “**Special-Place Deepening Technique”
**2**.**3**. **Therapeutic Suggestions**
**The Pain Switch** Informs patient about how the brain is the boss and the controller of the body, as everything is controlled by it. The patient is instructed to travel to the Brain’s Control Centre and visualize the “pain switch” *(e*.*g*. *“Your unconscious mind can also help you visualize this pain reception area*, *perhaps as a compartment or a master control room*. *When there is a lot of pain being reported there… you have a rheostat*, *a dimmer switch*, *which you can turn down… and experience less and less pain*.”). Make the patient see the nerve connection from the brain to the body area that is painful. Using out breath to begin turning the switch down with each breath and continue until the pain is low enough (*e*.*g*.*”Perhaps you will even want to rate it on a scale of 1 to 10*, *with 10 being the most pain that you would ever be in*. *And the number that you feel will be the number you can see in your mind and in this pain reception area…”*). The switch is then locked in that position (*e*.*g*. “…*so that you remain more comfortable and know you can help yourself in this way whenever you need*.*”)*.
**Direct Therapeutic Suggestions For Pain Control** Suggestions to change the pain transmission system using the Gate Control Theory of pain as a metaphor (*e*.*g*. *“… Your unconscious mind can relax all the nerves and muscle fibres in the area of your body where there is tension or pain… It can also interrupt the pathways which travel from the site of the injury*, *to the spinal cord… into the pain reception area*. *There are many*, *many gates which these pain impulses must pass through*, *and your unconscious mind can close many of these gates*, *reducing the number of nerve impulses that will finally reach the pain reception area*, *and so*, *you simply will be aware of less pain… Your brain is now sending messages to the gate-control stations to tune down the intensity and quality of the pain signals*, *so that you will feel less and less discomfort…”*). Suggestions to control or change pain perception, by providing a specific set of skills, such as relaxation, that could be used to alter how the brain processes pain information thus promoting pain relief (*e*.*g*. *“…as you allow yourself to go deeper and deeper*, *you are realizing*, *perhaps for the first time*, *that you can allow yourself to take control over your body through your mind*. *You can become more relaxed and with this relaxation your body’s healing mechanisms can function appropriately and normally*. *And as your body responds in this deeply relaxed way*, *you are becoming aware that you can permit yourself*, *when needed*, *to take greater control over your body than you ever thought possible*. *You are acquiring confidence in yourself… You are now in a relaxed state… An individual who is in such a relaxation state as you are does not feel pain as acutely as a person who is tense*. *In fact*, *sometimes*, *he feels no pain at all…*”).
**Ego Strengthening Technique**
**2**.**4**. **Post-Hypnotic Suggestions**
**3**. **Waking up**
**SESSION III & IV**
**1**. **Theoretical-Educative Contents**
**2**. **Hypnotic Techniques**
**2**.**1**. **Hypnotic Induction**: “Hartland’s Progressive Relaxation”
**2**.**2**. **Deepening Procedure: “**Special-Place Deepening Technique”
**2**.**3**. **Therapeutic Suggestions: “**Pain Switch”, “Direct Therapeutic Suggestions for Pain Control” and “Ego Strengthening Technique”
**2**.**4**. **Post-Hypnotic Suggestions**
**3**. **Waking up**

### Control group

Patients in the control group received medical treatments and standard care as usual, with the assessments being made in the same time points as the intervention group, but without receiving any psychological intervention.

### Outcomes and data collection

Feasibility and acceptability were evaluated by analysis of recruitment and retention rates, adherence to study protocol, and satisfaction with the intervention. The latter was assessed using four questions specifically developed for this study. These ask about overall satisfaction with the intervention (rated from 1 = very dissatisfied to 5 = very satisfied), perception of improvement in well-being after attending the four sessions (rated from 1 = nothing to 5 = totally), perception of the utility of this type of interventions in haemophilia centres and desire to participate in further sessions (both rated from 1 = disagree to 3 = agree).

To evaluate the effectiveness of the intervention, the following primary outcomes were considered: pain experience (intensity and interference) and HRQoL. To ensure the quality of self-reported data and avoid inter-assessor subjectivity, all assessments were performed in-person, by the same investigator, using the Portuguese version of the following measures:

- Socio-demographic and clinical questionnaire: Collects sociodemographic (*e*.*g*. age, education, employment) and haemophilia-related information (*e*.*g*. type and severity, bleeding episodes, treatment regimen).
- Multidimensional Haemophilia Pain Questionnaire (MHPQ)^66^: Assesses haemophilia-related pain, focusing on painful locations, duration, frequency, triggering factors, intensity, interference, strategies for pain control, pain specialists and satisfaction with treatment. Pain intensity [Cronbach’s alpha (α) = 0.65] is assessed for six triggers/situations: bleeding episodes; during physical efforts and/or movement; using stairs; after resting or staying still; during rest, sitting or lying down; and accidental or “wrong” movements. Interference (α = 0.82) is rated in 7 domains, using the Brief Pain Inventory subscale^67^ (general activity, mood, walking ability, normal work, relations with others, sleep and enjoyment of life). Pain intensity and interference items are rated on a numerical rating scale, ranging from 0 (no pain/no interference) to 10 (worst imaginable pain/completely interferes), with a mean score being computed for each scale.
- EQ-5D-5L^68^: Participants rate their overall HRQoL on five dimensions (mobility, self-care, usual activities, pain/discomfort and anxiety/depression) according to a five level scale (1 = no problems; 5 = extreme problems), with a summary index being calculated for each participant (range: 0–1; where 1 is the best possible score) (α = 0.58). Overall health status is evaluated by patients on a 0–100 visual analogic scale (VAS), with higher scores indicating a better health status.
- A36 Hemofilia-QoL^69^: This is a disease-specific measure to assess haemophilia-related quality-of-life. The 36 items are rated on a 5 point Likert scale, generating a global HRQoL score (possible range: 0–144; α = 0.90) and nine subscale scores (physical health, daily activities, joints, pain, treatment satisfaction, treatment difficulties, emotional functioning, mental health and relationships and social activity). Higher scores translate better HRQoL.
- Hospital Anxiety and Depression Scale (HADS)^70^: Used to evaluate emotional distress, according to two separate anxiety (α = 0.61) and depression (α = 0.92) scores (ranging from 0–21). Items are rated on a 4 point Likert scale (0–3), with higher results translating more symptomatology, and a score >8 being indicative of clinically significant symptoms^71^.

### Randomisation

Participants were randomly allocated to intervention or control group (1:1 ratio), with the different randomisation steps being performed independently by two investigators. The randomisation sequence was computer-generated and concealed until official patient enrolment, after consenting to participate and performing baseline assessment. Due to the differences in procedure, blinding of the patients to intervention *versus* control group was impossible. The clinicians from the haemophilia centre and the investigator performing the assessments were blind to patient allocation.

### Data analysis

Data analyses were performed using IBM SPSS Statistics version 23 (Chicago, IL, USA). Participants’ characteristics are expressed as absolute and relative frequencies (n, %) for categorical data or mean and standard deviation (SD) for continuous variables.

To adjust for between-group baseline differences, changes from pre to post-test assessment were analysed by calculating the difference between the two moments (Δ = T1 score-T2 score). The normality assumption of the Δ variables was checked for the two groups (CG and EG) through Shapiro-Wilk test. Normality was guaranteed for all variables, except for “number of bleeds” and “missed work days” in both groups; and for the “joints”, “pain” and “relationships and social activity” subscales of the A36 Hemofilia QoL, in the EG. For these cases, the corresponding non-parametric tests were used, and since the results were concordant with the corresponding parametric procedures, we decided to report the latter.

The groups were compared through chi-squared tests (χ2, for categorical variables) or independent-sample t tests (for continuous variables), accounting for the equality of variances (Levene’s test). If the variances were not homogeneous, the Welch’s correction was used. Statistical significance was set at p < 0.05.

Since null hypothesis significance testing and, consequently, p values, depend on sample size, the meaningfulness of differences was determined through the associated effect sizes (ES). These were expressed as Cohen’s *d* for continuous variables and Pearson’s phi (φ) coefficient for nominal variables. Cohen’s *d* score above 0.80 is considered a large effect, between 0.50 and 0.80 a medium effect and between 0.20 and 0.49 a small effect^72^. The interpretation of Pearson’s phi (φ) coefficient is analogous to the correlation coefficient, expressing the strength of association between two variables. The internal consistency of responses to the questionnaires was assessed using the Cronbach’s alpha coefficient (α)^73^.

## Data Availability

The datasets generated during and/or analysed during the current study are available from the corresponding author on reasonable request.
